# Extending the intermedullary nail will not reduce the potential risk of femoral head varus in PFNA patients biomechanically: a clinical review and corresponding numerical simulation

**DOI:** 10.1186/s12891-024-07334-z

**Published:** 2024-05-23

**Authors:** Xiaofeng Cha, Qin Zhou, Jujie Li, Hong Xu, Wenqiang Xu, Jingchi Li

**Affiliations:** 1 Department of Orthopaedics, Luzhou Hospital of Traditional Chinese Medicine, Luzhou, Sichuan Province 646000 P.R. China; 2 Department of Orthopedics, Luzhou Key Laboratory of Orthopedic Disorders, The Affiliated Traditional Chinese Medicine Hospital, Southwest Medical University, Luzhou, Sichuan Province P.R. China; 3 Department of Orthopaedics, Affiliated Hospital of Integrated Traditional Chinese and Western Medicine, Nanjing University of Chinese Medicine, Nanjing, Jiangsu Province 210028 P.R. China

**Keywords:** Intermedullary nail length, PFNA, Femoral head varus, Case-comparative study, Finite element analysis

## Abstract

Femoral head varus is an important complication in intertrochanteric fracture patients treated with proximal femoral nail anti-rotation (PFNA) fixation. Theoretically, extending the length of the intramedullary nail could optimize fixation stability by lengthening the force arm. However, whether extending the nail length can optimize patient prognosis is unclear. In this study, a review of imaging data from intertrochanteric fracture patients with PFNA fixation was performed, and the length of the intramedullary nail in the femoral trunk and the distance between the lesser trochanter and the distal locking screw were measured. The femoral neck varus status was judged at the 6-month follow-up. The correlation coefficients between nail length and femoral neck varus angle were computed, and linear regression analysis was used to determine whether a change in nail length was an independent risk factor for femoral neck varus. Moreover, the biomechanical effects of different nail lengths on PFNA fixation stability and local stress distribution have also been verified by numerical mechanical simulations. Clinical review revealed that changes in nail length were not significantly correlated with femoral head varus and were also not an independent risk factor for this complication. In addition, only slight biomechanical changes can be observed in the numerical simulation results. Therefore, commonly used intramedullary nails should be able to meet the needs of PFNA-fixed patients, and additional procedures for longer nail insertion may be unnecessary.

## Introduction

Femoral intertrochanteric fracture is the most common hip fracture in elderly patients [1, 2]. Although hip arthroplasty is an alternative treatment strategy, internal fixation is still the most commonly used surgical method for these patients [1, 3]. Proximal femoral nail anti-rotation (PFNA) fixation has been widely used to treat intertrochanteric fractures and has achieved a credible clinical prognosis in most patients [4, 5].

The optimal length of the main nail has not been determined during PFNA fixation. Several studies have shown that long nails were developed to reduce the potential risk of stress concentration and resulting complications by extending the load arm of the intermedullary nail [6, 7]. However, several recent studies have shown that the use of longer nails does not obviously reduce the potential risk of complications [8–10]. In addition, extending the nail length significantly increases blood loss, surgical time, and even intraoperative radiography [7, 10]. Therefore, the absence of existing guidelines and the treating surgeon’s preference, rather than any objective factors, influence the decision of nail length.

Postoperative femoral head varus was still a commonly observed complication in patients who underwent PFNA fixation [4, 11], which adversely affects patient recovery. As an important [12]intraoperative parameter, the length of the nail significantly affects the instant postoperative local biomechanical environment (i.e., fixation stability and stress distribution on the femoral head) [6]. Given that deterioration of the biomechanical environment could initially trigger femoral head varus, the nail length of PFNA may potentially affect the risk of this complication. However, to our knowledge, this topic has yet to be identified.

In this study, by reviewing the clinical data of PFNA-fixed patients, the clinical effect of nail length on the incidence of femoral head varus was investigated, and the corresponding biomechanical mechanism of clinically observed phenomena was also verified via numerical simulations. Identifying this topic could provide theoretical foundations for the optimization of intraoperative procedures and corresponding clinical prognoses for PFNA-fixed patients.

## Materials and methods

### Clinical review

#### Patient collection

**Fig. 1:**
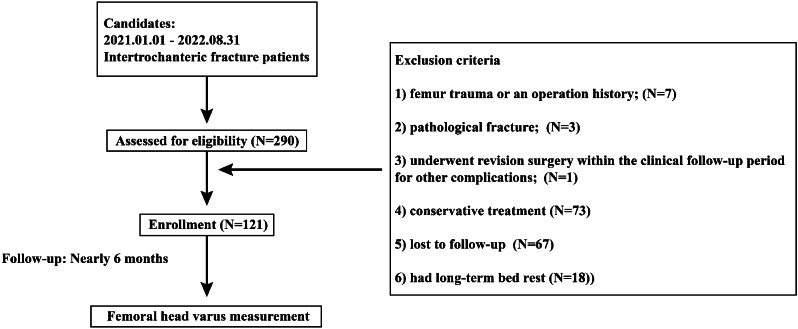
Schematic for patient inclusion and exclusion

The ethics committees of our hospital reviewed and approved the protocol of this study. Informed consent was waived for this retrospective study. We retrospectively reviewed patients who suffered from the intertrochanteric fractures and underwent PFNA fixation from January 2021 to August 2022. The exclusion criteria were as follows: (1) had femur trauma or an operation history; (2) had a pathological fracture caused by primary or metastatic bone tumors, bone tuberculosis, or rheumatic immune diseases; (3) underwent revision surgery within the clinical follow-up period of 6 months for other complications; (4) had conservative treatment; (5) were lost to follow-up or died during the follow-up cycle; and (6) had long-term bed rest (larger than 2 weeks) (Fig. 1) [13, 14]. Patients baseline characteristics, including age, sex, and BMI, were recorded [15, 16]. A well-trained orthopedic surgeon performed all the PFNA operations, and the tip-to-apex distance (TAD) for the anti-rotation blade on the anterior-posterior radiography was < 25 mm, and the blade was located in the inferior half of femoral head for all the enrolled patients [4, 17]. Patients with stable fracture type began weight-bearing on the injured limb (walking on the ground) within 3 days after surgery, and patients with unstable fracture began injured weight-bearing within 2 weeks.

#### Radiographic data collection

**Fig. 2:**
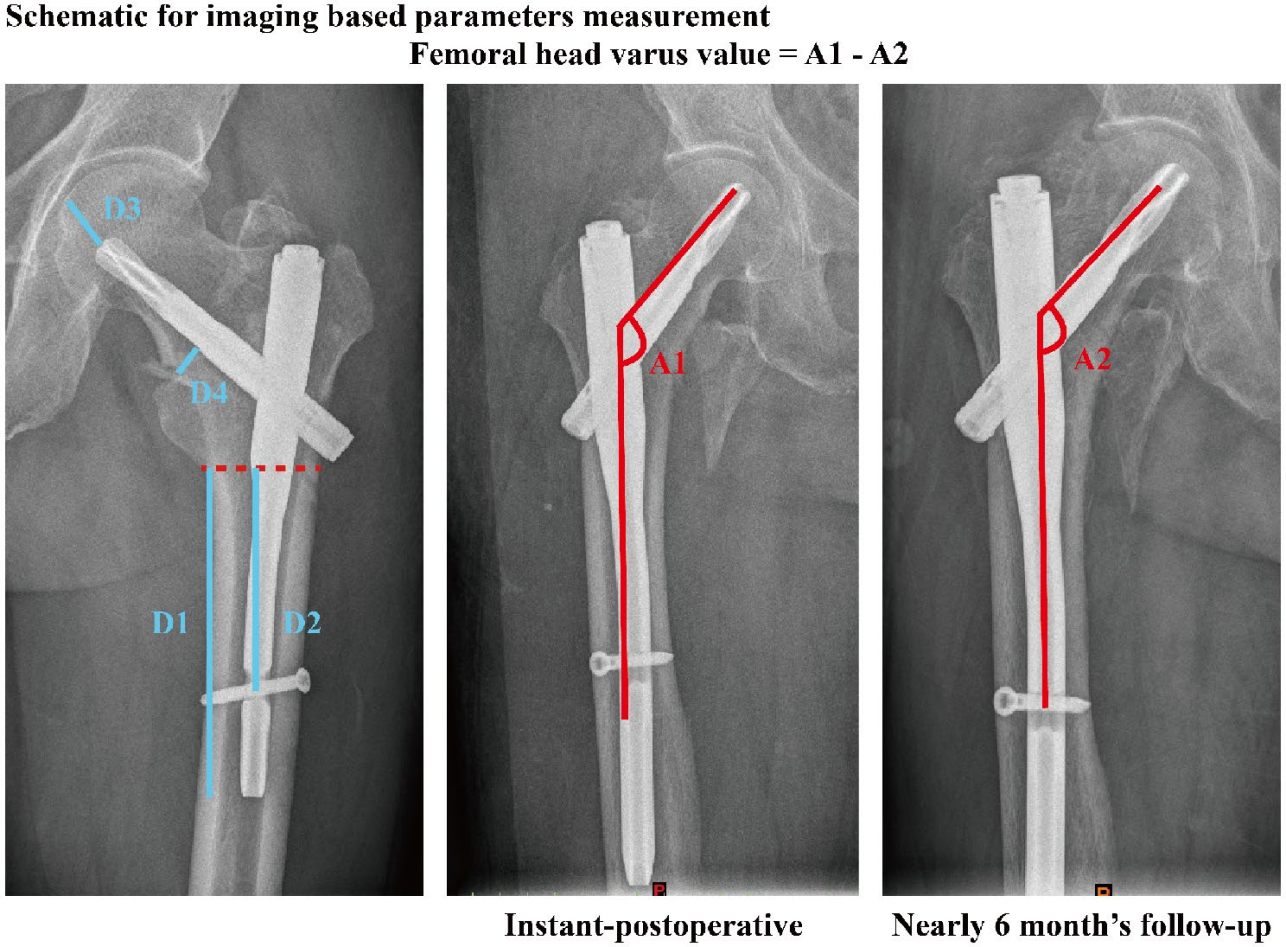
The measurement of imaging parameters: The length of the intramedullary nail in the femoral trunk (D1), the distance between the lesser trochanter and the distal locking screw (D2), and the femoral head varus values, TAD in the anterior-posterior radiography (D3), and the distance between femoral calcar and the anti-rotation blade (D4)

All patients underwent anterior-posterior radiography three times, including immediately before and after the operation and nearly 6 months after the screw fixation operation. The femoral neck-trunk angle was measured via anterior-posterior radiography at three different times, and the difference between pre and instant postoperative angles was computed to represent the varus correlation status, and that between instant postoperative and 6 moths’ follow up was computed to represent the femoral head varus progression status [4, 18]. The length of the intramedullary nail in the femoral trunk (below the lesser trochanter plane) and the distance between the lesser trochanter and the distal locking screw were measured separately (Fig. 1). Fracture type was defined in the preoperative anterior-posterior radiography. AO 3.1 A1.1, 1.2, 1.3 and 2.1 types fracture was defined as “stable fracture”, AO 2.2 and 2.3 types fracture was defined as “unstable fracture”. TAD value and the distance between femoral calcar and anti-rotation blade on the central point of femoral neck were measured in the instant postoperative anterior-posterior radiography (D3 and D4, Fig. 2).

### Statistical analyses

We conducted the statistical analyses with SPSS software. One week after completing the radiological data measurements, a randomly selected imaging specialist with extensive experience in orthopedic imaging and the aforementioned orthopedic physician re-evaluated the imaging data for 20 patients to assess interrater reliability. Intraclass correlation coefficients (ICCs) and Kappa value were calculated to determine the consistency of continue and binary variables [19, 20]. Excellent interrater reliability was judged when the ICC and Kappa value was > 0.8 [19, 21]. Normality tests for continuous variables were performed, and Pearson and Spearman correlation coefficients between normally distributed continuous variables, nonnormally distributed continuous variables and categorical variables and femoral head varus values were calculated separately [22, 23]. Moreover, we performed linear logistic regression to identify independent risk factors for femoral head necrosis. Univariate analyses of each potential risk factor were performed, and the variables that achieved a significance level of *p* < 0.1 were entered into multivariate analyses. Variables with *P* < 0.05 were considered independent risk factors in the multivariate analysis [24, 25].

### Numerical surgical simulations and finite element analyses (FEA)

#### Construction of the intact finite element (FE) model

The proximal femur model was constructed based on the outline of the syn-bone model rather than that of any special patient. The model construction strategy was selected to avoid ethics-related procedures and eliminate the confounding effects caused by individual differences in the outlines of different patients [18, 26]. A thin CT scan was performed on the syn-bone femur model (thickness = 0.55 mm). The range of the proximal femur was defined as the distance from the tip of the femoral head to 30 cm below the lesser trochanter. The outline of the proximal femur model was constructed according to the CT-scanned femur outline in 3D-CAD software [27, 28]. This study consisted of our published studies. The computational efficiency and accuracy of the numerical model constructed by this method were better than those of the traditional reverse model construction strategy [15, 29]. Cortical and cancellous bones were separately constructed, and outlines of these bony structures were constructed separately based on the CT imaging data [30, 31].

#### Construction of PFNA fixed intertrochanteric fracture models with different nail length

**Fig. 3:**
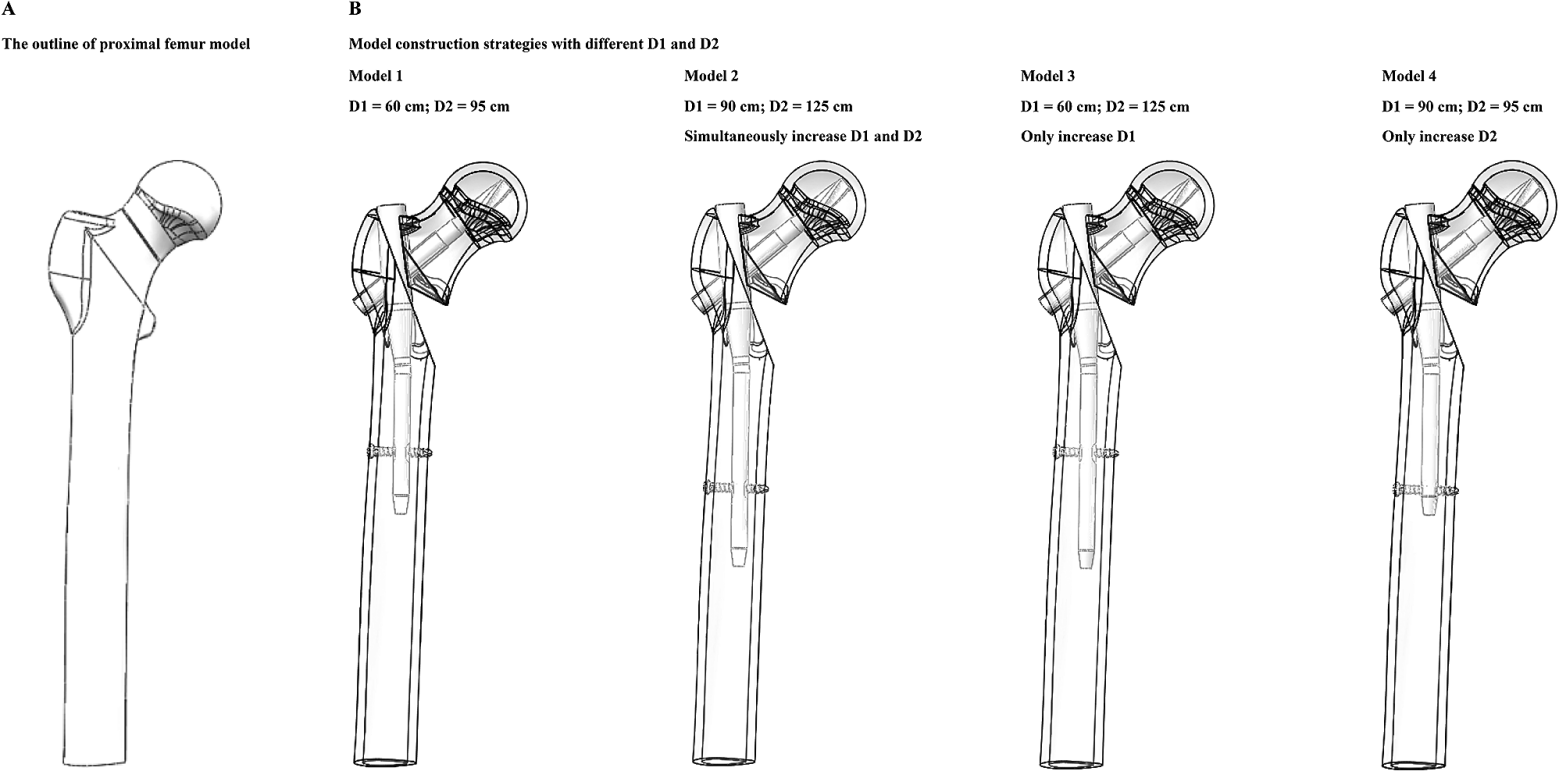
Schematic for numerical model construction, and surgical simulations in models with different length of the intramedullary nail in the femoral trunk, and different distances between the lesser trochanter and the distal locking screw

To simulate the fixation stability of different screw fixation strategies in the instability fracture model, the AO 3 − 1 2.3 type intertrochanteric fracture model was constructed [26, 32]. The specific modeling method involved creating the fracture by intersecting three fracture lines. The first fracture line was positioned 10 mm below the greater trochanter, forming a 20° angle with the long axis of the femoral shaft [18, 26]. The second fracture line was set tangent to the upper edge of the lesser trochanter, and the third fracture line connected the intersection of the first and second lines with the vertex of the greater trochanter. The bone within the cut range was removed to complete the reconstruction of the fracture model. The numerical model of a PFNA fixation device was also constructed with 3D-CAD software [30, 32]. When simulating the PFNA fixation operation, the trajectory of the anti-rotation blade was coaxial to the long axis of the femoral neck [11, 18]. By adjusting the nail length and the position of the distal locking screw, the biomechanical significance of changes in clinically observed parameters (i.e., the length of the intramedullary nail in the femoral trunk and the distance between the lesser trochanter and the distal locking screw) has been investigated via numerical models. The detailed model construction strategy is presented in Fig. 3.

#### Boundary and loading conditions

**Fig. 4:**
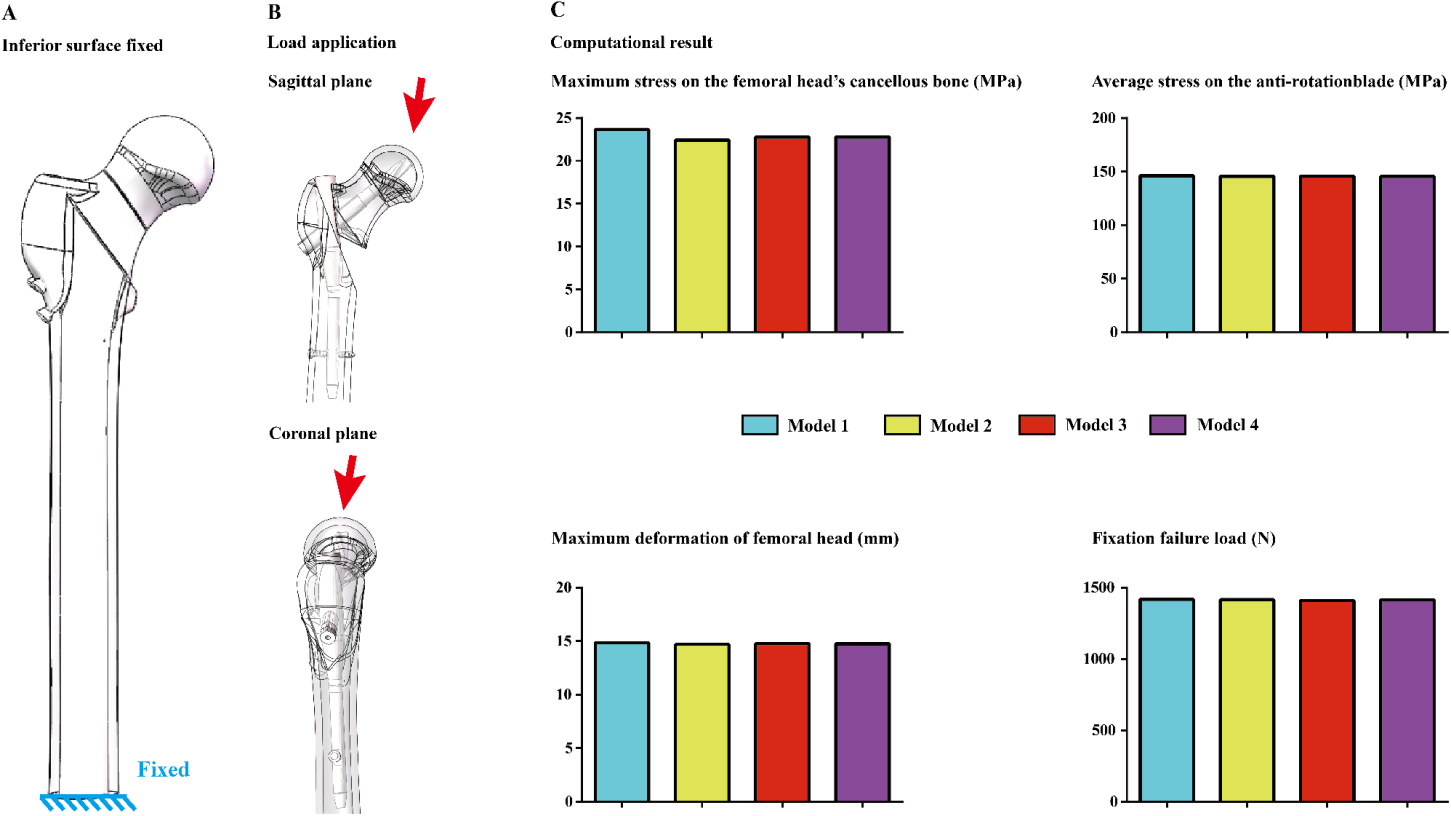
Boundary and loading conditions for numerical simulation, computational results in different models

The material properties of cortical and cancellous bone and the titanium alloy cannulated screw were separately defined as isotropic material according to the same type of study. The degrees of freedom of the inferior surfaces of the numerical models were completely fixed. Different forces were applied to the femoral head at 10° lateral to the coronal plane and 9° posteriorly to the sagittal plane [3, 33]. Two different loading protocols were applied on the superior surface of the femoral head. A 2100 N load was applied on the femoral head. The stress distributions of the femoral head and anti-rotation blade, the stress distributions of the femoral head and anti-rotation blade, the maximum displacement of the femoral head, and the failure load resulting from fixation failure were computed and recorded to determine the potential risk of femoral head varus (Fig. 4) [30, 34].

## Results

### Clinical review and judgement of independent risk factors for femoral head varus in PFNA fixed patients

Excellent intra- and interobserver measurements of imaging-based parameters were judged based on the computed ICC and Kappa values (Table 1). In this study, clinical data from 121 patients were collected. Correlation analysis indicated no significant correlation between nail length parameters (i.e., the nail length in the femoral trunk or the distance between the lesser trochanter and the distal locking screw) and the incidence of femoral head varus, and these parameters were also not found to be independent risk factors for femoral head varus. Besides, larger TAD value and unstable type of fracture were significantly correlated to femoral head varus, but only unstable fracture was an independent risk factor for femoral head varus progression. Meanwhile, the distance between femoral calcar and blade was not significantly correlated to femoral head varus values, and was not an independent risk factor for femoral head varus progression. Moreover, there were no significant difference between D1 and D2, and the femoral head varus correlation status (Tables 2, 3 and 4). Finally, no patients suffered diaphyseal fractures in the absence of additional trauma during the clinical follow-up period. Fixation strength computation.

**Table 1:** ICC and Kappa values of inter- and intraobserver reliability when measuring imaging based parameters

	Intra-observer	Inter-observer
Femoral head varus progression	0.889	0.91
The length of the intramedullary nail in the femoral trunk (D1)	0.913	0.859
The distance between the lesser trochanter and the distal locking screw (D2)	0.902	0.838
Femoral head varus correlation	0.882	0.873
Fracture types	0.894	0.894
TAD in the anterior-posterior radiography (D3)	0.827	0.876
Distance between femoral calcar and blade (D4)	0.865	0.882

**Table 2:** Correlation coefficients between femoral head varus and variates

Femoral head varus	Correlation coefficients	P value
Baseline characteristics		
Age	-0.107	0.243
Sex (Male: 1, Female: 2)	0.106	0.259
BMI	-0.097	0.287
Imaging based parameters		
The length of the intramedullary nail in the femoral trunk (D1)	0.006	0.95
The distance between the lesser trochanter and the distal locking screw (D2)	-0.035	0.699
Fracture types	0.259	0.004**
TAD in the anterior-posterior radiography (D3)	0.202	0.026*
Distance between femoral calcar and blade (D4)	-0.004	0.964

*, statistical significance (*P*<0.05)

**, statistical significance (*P*<0.01)

**Table 3:** Linear regression analysis of severe femoral head varus

	t	95% CI		P value
Uni-variable analyses				
Baseline characteristics				
Age	-1.174	-0.11	0.028	0.243
Sex (Male: 1, Female: 2)	0.622	-1.042	1.996	0.535
BMI	-1.069	-0.532	0.159	0.287
Imaging based parameters				
The length of the intramedullary nail in the femoral trunk (D1)	0.063	-0.052	0.056	0.95
The distance between the lesser trochanter and the distal locking screw (D2)	-0.388	-0.086	0.058	0.699
Fracture types	2.682	0.462	3.067	0.008#
TAD in the anterior-posterior radiography (D3)	2.26	0.017	0.262	0.026#
Distance between femoral calcar and blade (D4)	-0.045	-0.236	0.226	0.964
Multi-variable analyses				
Fracture types	2.32	0.225	2.852	0.022*
TAD	1.829	-0.009	0.236	0.07

^#^,variables that achieved a significance level of *p* < 0.1 in the univariate analysis

*, statistical significance in multi-variable analyses (*P*<0.05)

**Table 4:** Correlation coefficients between instant postoperative femoral head varus correlation and variates

Femoral head varus	Correlation coefficients	P value
The length of the intramedullary nail in the femoral trunk (D1)	0.016	0.865
The distance between the lesser trochanter and the distal locking screw (D2)	-0.028	0.761

*, statistical significance (*P*<0.05)

**Fig. 5:**
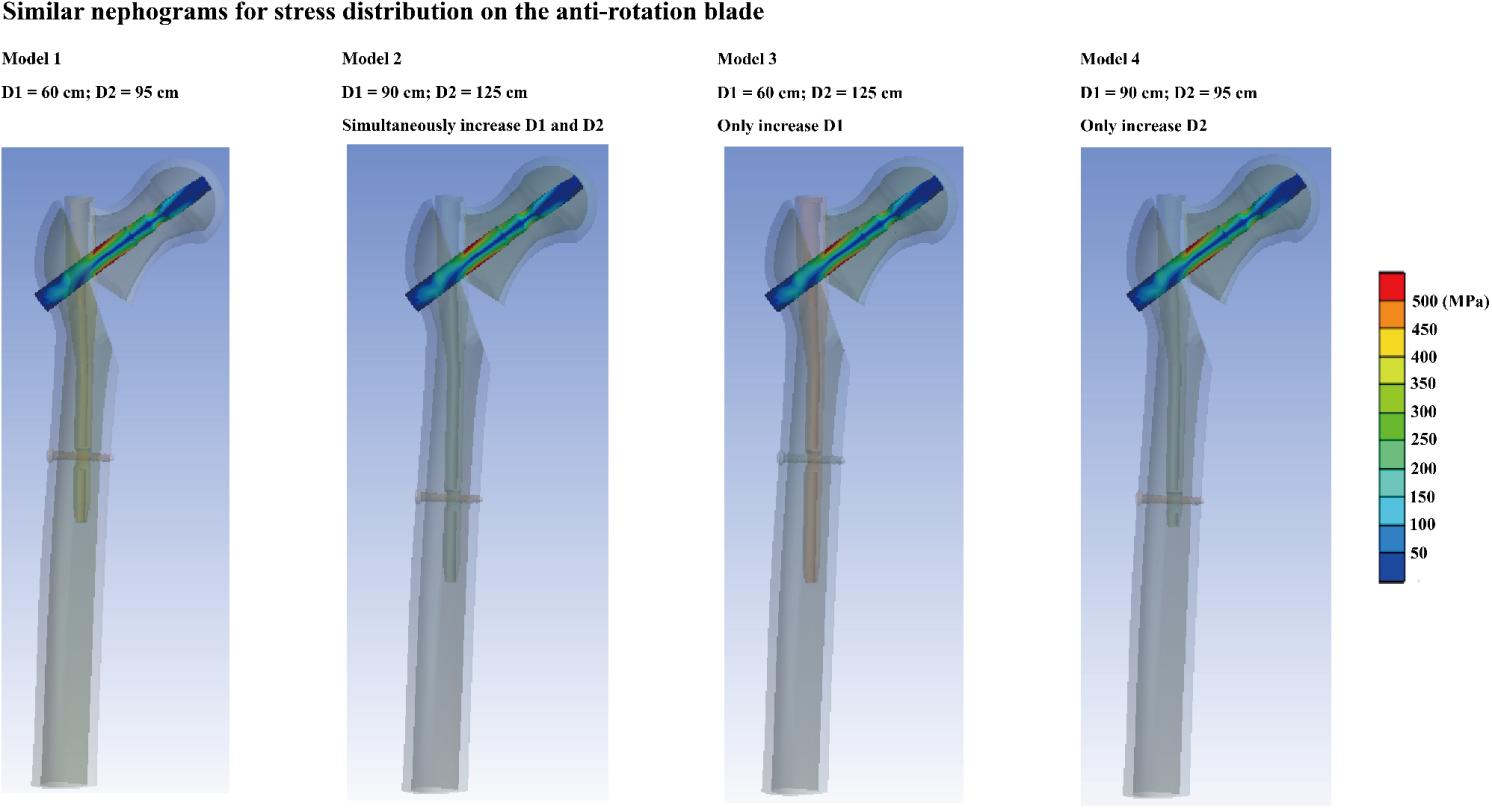
Nephograms for anti-rotation blade stress distributions: Slight difference in stress distribution can be observed in different numerical models

Consistent with the results of previous reviews, a consistent variation in the computed parameters can be observed in the current numerical models. Specifically, in the model simultaneously extending nail length in the femoral trunk and the distance between the lesser trochanter and the distal locking screw, the maximum stress of cancellous bone reduced by more than 5%, differences in other parameters was less than 1% between different models. Moreover, contrary to our expectations, compared to those in the control group, the fixation failure loads in the nail length extension and the distance between the lesser trochanter and the distal locking screw groups were slightly lower (Figs. 4 and 5).

## Discussion

Theoretically, extending the intermedullary nail in PFNA-fixed patients can optimize fixation stability by alleviating local stress concentrations [12, 35]. This approach may reduce the risk of biomechanical complications, especially diaphyseal fractures [6, 7]. However, comparable prognoses have also been reported for patients with short or long nails. More significantly, extending the nail length can trigger an increase in blood loss, surgical time, and even intraoperative radiography examination time and inhibit patients’ quick recovery [6, 7]. Moreover, although there were differences in indications between “short and long nail” selection (e.g., long nail was more suitable for patients with subtrochanteric fracture) [6, 7], and all PFNA used in the current patients were traditional “short nail”. However, the effect of nail length changes on fixation stability and corresponding risk of femoral head varus has yet to be identified in patients with “short nail” PFNA fixation.

To verify this topic and provide theoretical and practical guidance for the optimization of PFNA operation, we performed comprehensive research consisting of a clinical review and corresponding numerical simulations. A consistent variation tendency can be observed in this study. That is, extending the nail length will not alleviate the stress concentration on the femoral head or the corresponding risk of femoral head varus. Therefore, based on the current results, commonly used intramedullary nail lengths should be able to meet the needs of PFNA-fixed patients, and additional iatrogenic injury caused by longer nail insertion should be avoided. In addition, although studies have reported that extending the nail length can reduce the risk of postoperative diaphyseal fracture, no patients in the present study experienced this complication. This is mainly due to the relatively high strength of the cortex in the diaphysis and the lower activity in elderly patients with intertrochanteric fractures.

Moreover, although larger TAD value was significantly correlated to femoral head varus, this factor was not an independent risk factor for femoral head varus progression. This result indicate that in patients whose TAD < 25 mm, the influence of TAD value on fixation stability was limited, and this was consisted to published studies [4, 36]. Moreover, studies present that femoral calcar is an important structure to provide the medium support of the femur [1, 37], but the current study indicate that the distance between calcar and anti-rotation blade was not significantly affect the risk of femoral head varus progression. Therefore, we can also deduce that place the blade in the inferior part of femoral neck can construct comparable fixation stability in PFNA operation, but the detail relation between blade position and PFNA fixation stability still should be verified in our future studies.

The following topics from the methodological perspective should be clarified in this study. First, femoral head varus values were recorded to determine patient prognosis. The reoperation rate of PFNA-fixed patients was recorded to determine the complication rate in several studies [9, 10]. Traditionally, only patients who underwent reoperation were considered to suffer complications [10, 38]. However, studies have shown that even in patients who do not undergo reoperation, the progression of femoral head varus can also trigger clinical deterioration, even in patients with fracture union [39, 40]. Therefore, this study evaluated patient prognosis by measuring the femoral head varus angle and can provide a new perspective for optimizing the PFNA operation.

In addition, although mechanical tests can directly reflect fixation stability in models with internal fixation [41, 42], numerical models were selected to determine the biomechanical mechanism of changes in the PFNA strategy in this study. The fresh specimen model-based mechanical test can only reflect the overall fixation stability change, and numerical simulations can directly reflect the stress distribution on the blade-bone interface [24, 43]. More significantly, numerical mechanical simulation can discretize the biomechanical significance of particular variables [15, 16]. In this study, the biomechanical significance of nail length extension and the distance between the lesser trochanter and the distal locking screw were separately investigated, but these factors are not feasible for mechanical tests. Meanwhile, when investigating the effect of a certain variable on the fixation stability, researchers prone to construct numerical models with unstable type of fracture (e.g., A.2.3 fracture type in this study), this was a commonly used model construction strategy on the same type studies [18, 26, 32]. That’s because this model construction strategy can reduce potential incidence of false negative result.

The combination of clinical review and mechanical simulation can optimize the credibility of this study [15, 16]. As a study with negative results (i.e., changes in nail length will not significantly affect the risk of femoral head varus) and if only the clinical review section was involved in this study, researchers may consider that the limited sample size may lead to potential false negatives. Similarly, if the significance of nail length changes has been investigated by numerical simulations alone, researchers may consider that improper model construction strategies or loading conditions can trigger false negative results. In contrast, when negative clinical review results and computational results can be mutually corroborated, the reliability of the study results can be effectively guaranteed. Finally, the syn-bone model was selected to construct the outline of the numerical model. Although this modeling strategy suffers from defects (simplification of cortical and cancellous bony regions in the standard model and the outline of certain anatomical structures) [18, 32], it has been widely used for its inherent advantages, such as the nonrequirement of an ethical review and repeatable results (elimination of confounding effects of patient-specific morphological parameters on computational) [18, 26].

Admittedly, this study has the following limitations. First, only one fracture type was selected to construct the numerical model. Changes in fracture type, especially different grades of medial or lateral wall injury, and the interactions between these parameters and nail length changes should be verified in our future studies [1, 44]. In addition, different trabecular structures, including compressive and tensile trabecular structures, play significant biomechanical roles in maintaining PFNA fixation stability [11, 13]. Due to the lack of a standard model construction strategy, these structures have been simplified to iatrogenic structures in current numerical models. Moreover, limited by the lack of fatigue test related mechanical material properties (e.g., s-n curve), only one cycle of load was applied on the numerical model. As a result, biomechanical significance during the cyclic load can not be identified in this study, and this was an important limitation of the current study. However, as a comprehensive research consisted by clinical review and numerical simulations, an identical tendency can be observed in both two parts of this study. Therefore, we believe that this limitation will not significantly affect the credibility of the current research conclusion.

Meanwhile, the ignorance of interaction between blade trajectory on the coronal and transverse planes was also a main limitation of this study [6, 45]. However, for the lack of instant postoperative CT imaging data, the blade trajectory on the transverse plane can not be directly judged, and which should be identified in our future studies. Finally, although this study revealed that commonly used intramedullary nails should be able to meet the needs of PFNA-fixed patients, according to the basic principle of biomechanics, we believe that the reduction in nail length is not unlimited and that a safe threshold should be identified in our future biomechanical studies.

## Data Availability

All data generated or analysed during this study are included in this published article and its supplementary information files.
